# Time-resolved photoluminescence on double graded Cu(In,Ga)Se_2_ – Impact of front surface recombination and its temperature dependence

**DOI:** 10.1080/14686996.2019.1586583

**Published:** 2019-04-09

**Authors:** Thomas Paul Weiss, Romain Carron, Max H. Wolter, Johannes Löckinger, Enrico Avancini, Susanne Siebentritt, Stephan Buecheler, Ayodhya N. Tiwari

**Affiliations:** a Laboratory for Thin Films and Photovoltaics, Empa – Swiss Federal Laboratories for Materials Science and Technology, Dübendorf, Switzerland; b Laboratory for Photovoltaics, Physics and Materials Science Research Unit, University of Luxembourg, Belvaux, Luxembourg

**Keywords:** Time-resolved photoluminescence, Cu(InGa)Se_2_, trapping, minority carrier lifetime, bandgap grading, 50 Energy Materials, 209 Solar cell / Photovoltaics, 505 Optical / Molecular spectroscopy

## Abstract

Time-resolved photoluminescence (TRPL) is applied to determine an effective lifetime of minority charge carriers in semiconductors. Such effective lifetimes include recombination channels in the bulk as well as at the surfaces and interfaces of the device. In the case of Cu(In,Ga)Se_2_ absorbers used for solar cell applications, trapping of minority carriers has also been reported to impact the effective minority carrier lifetime. Trapping can be indicated by an increased temperature dependence of the experimentally determined photoluminescence decay time when compared to the temperature dependence of Shockley–Read–Hall (SRH) recombination alone and can lead to an overestimation of the minority carrier lifetime. Here, it is shown by technology computer-aided design (TCAD) simulations and by experiment that the intentional double-graded bandgap profile of high efficiency Cu(In,Ga)Se_2_ absorbers causes a temperature dependence of the PL decay time similar to trapping in case of a recombinative front surface. It is demonstrated that a passivated front surface results in a temperature dependence of the decay time that can be explained without minority carrier trapping and thus enables the assessment of the absorber quality by means of the minority carrier lifetime. Comparison with the absolute PL yield and the quasi-Fermi-level splitting (QFLS) corroborate the conclusion that the measured decay time corresponds to the bulk minority carrier lifetime of 250 ns for the double-graded CIGS absorber under investigation.

## Graphical Abstract

**Figure:**
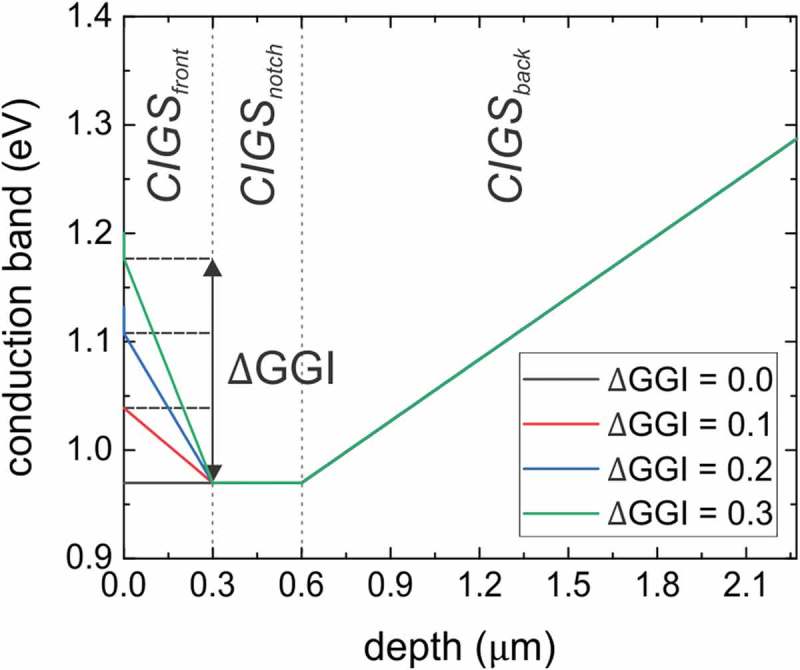

## 1. Introduction

Time-resolved photoluminescence (TRPL) is generally applied to measure the minority carrier lifetime of Cu(In,Ga)Se_2_ (CIGS)-based p-type semiconducting absorbers and a good correlation to device efficiency is observed [1–5]. However, experimental evidence has been presented that trapping of minority charge carriers may impact these measured lifetimes, which results in measured effective lifetimes larger than the bulk lifetime [6,7]. These trapping states are incorporated into several simulation models presented in literature to describe experimental data [8,9]. The main experimental evidence for trapping is a temperature dependence of the measured photoluminescence (PL) decay times, which cannot be explained by the temperature dependence of Shockley–Read–Hall (SRH) recombination [7]. Redinger et al. showed that a degradation of the absorbers surface might also cause a strongly temperature dependent PL decay time, which can be suppressed when measuring in N_2_ atmosphere instead of ambient air [10] and the authors concluded that trapping is not present in their CIGS absorber.

In this manuscript, the temperature dependence of the measured PL lifetimes is analyzed experimentally and corroborated by simulations. A double-graded absorber is considered, i.e. an absorber, with a bandgap minimum within the bulk of the absorber. Such a double-grading is generally obtained when growing the absorber using the three-stage process [11]. It is noted that a three-stage deposition process has also been used to grow the absorbers in other studies, who investigated the temperature dependence of the PL decay time [6,7,10]. We demonstrated that the temperature dependence of the PL decay time is well described by SRH statistics for well-passivated surfaces of the double-graded absorber. In contrast, for a non-passivated front surface, the PL decay time decreases more rapidly with increasing temperature. We used the PL decay time to calculate the external radiative efficiency as well as the quasi-Fermi-level splitting (QFLS). A good match to direct measurements of these quantities is observed indicating that the PL decay time is the minority carrier lifetime of the CIGS absorber.

## 2. Experimental

The CIGS absorber is grown using a multi-stage co-evaporation process from elemental sources on a Mo/SiO_x_/soda lime glass (SLG) substrate as detailed in Ref [12]. The growth is tailored to yield a [Ga]/([Ga]+[In]) (GGI) depth grading with a total thickness of approximately 3 μm as shown in Supplementary Figure 1. After the growth, an in situ post-deposition treatment with NaF and RbF has been applied [13]. Solar cell devices (stack of SLG/SiO_x_/Mo/CIGS/CdS/i:ZnO/Al:ZnO/Ni-Al grids) from a second absorber layer grown in the same deposition run yield a maximum and average (from 18 solar cells) efficiency without anti-reflective coating of 19.9% and 19.2%, respectively. The integral composition of the absorber was determined by X-ray fluorescence and the GGI grading by secondary ion mass spectrometry as detailed elsewhere [14].

**Figure 1.**
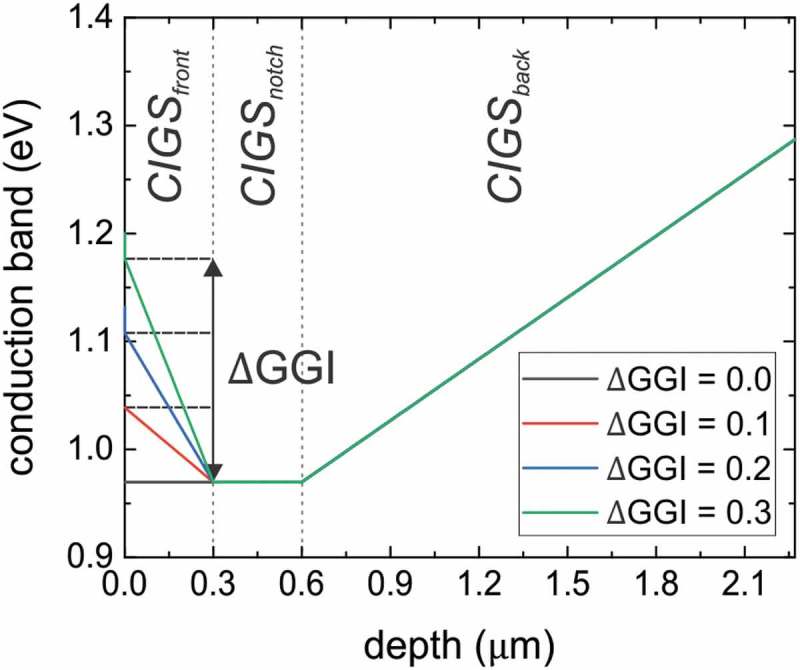
Modeling of the conduction band edge for a double graded CIGS absorber. While the CIGS_notch_ and the CIGS_back_ region were kept constant, the GGI increase toward the front surface was varied. The valence band was constant throughout the CIGS absorber. The energy E=0 eV corresponds to the Fermi level.

Temperature-dependent TRPL curves are measured using three different configurations of the front surface of the CIGS absorber, which lead to different front surface recombination velocities (see below). The first configuration consists of a chemical bath deposited CdS buffer layer (14 min deposition) of approximately 30-nm thickness (subsequently labeled: *14ʹ CdS*). The CdS buffer layer has been shown to yield stable TRPL measurements [15] with a rather low front surface recombination velocity [16]. For the second configuration, the CdS layer was removed by etching the absorber in 5 wt.% HCl for 1 min (subsequently labeled: *after HCl*). It is reported that the free CIGS surface degrades in ambient air [10,17] and thus leads to an increased front surface recombination velocity [16]. The third configuration was done by a 10 wt.% KCN etch for 3 min followed by a chemical bath deposited CdS buffer layer of roughly 19 min (subsequently labeled: *KCN, 19ʹ CdS*) of the HCl-etched absorber. The KCN etch was done to restore a high-quality surface after the degradation in air [17]. The deposition time of CdS was prolonged as a possible Rb-induced front surface layer might be (at least partly) removed by the HCl etch [13,18]. It is stressed that the three surface configurations were fabricated subsequently on the same piece of absorber.

TRPL measurements were carried out using a pulsed laser (pulse width ≈ 100 ps) on a spot size with a diameter of roughly 50 μm with a wavelength of 639 nm, i.e. the laser light is not absorbed in the CdS layer. The excitation density was around $6.5\times 10^{11}cm^{-2}pulse^{-1}$, which results in an electron density at the surface in the same order as the doping density of the CIGS absorber directly after the pulse. The PL emission is measured spectrally integrated. Additional information of the TRPL system can be found in [16]. The temperature of the absorber was varied by using a Peltier element below the sample. The temperature was monitored with a thermocouple glued on top of a SLG substrate, which was placed next to the absorber under test. TRPL measurements were taken on two temperature cycles for each sample to assess the reversibility of the observed temperature dependency of the PL decay time and to exclude modifications of the bulk and/or surface properties upon thermal treatment.

Steady-state PL measurements were carried out by exciting the sample with the 660 nm line of a diode laser on a spot size of 2.6 mm. The measurements were calibrated for absolute values by using a commercial spectralon that mimics the lambertian reflectance/emittance of the sample surface. The incident photon flux density from the laser was calibrated to the equivalent flux density of the AM1.5 sun spectrum above a bandgap energy of 1.1 eV i.e. 2.76 · 10^17^ photons cm^−2^ s^−1^. The emitted PL light from the sample was collected by two off-axis parabolic mirrors and redirected into a 303 mm focal length spectrometer, where it was dispersed and detected by a 512 element InGaAs array. A commercial calibrated halogen lamp was used for spectral correction.

One-dimensional simulations of TRPL decays were carried out using the Synopsys technology computer-aided design (TCAD) software by solving the Poisson equation and continuity equation for electron and holes. The transport is described by drift-diffusion equations. The CIGS absorber is modeled by three regions CIGS_front_, CIGS_notch_ and CIGS_back_ as shown in Figure 1. Various configurations of the CIGS_front_ region were explored, but the CIGS_notch_ and the CIGS_back_ regions were not changed for the simulations presented in this study. The conduction band increase toward the front contact has been varied by changing ΔGGI. The impact of the ΔGGI has been modeled by a variation of the bandgap via $E_{g}=1.01+0.69\times GGI$ as well as a change in the electron affinity by ΔX=−0.69×GGI (i.e. a simplified relation without a bowing factor). Consequently, only the conduction band is varied, while the valence band is flat throughout the absorber (not shown in Figure 1). Bulk SRH recombination has been simulated using the SRH recombination keyword in the Sentaurus TCAD physics section including the temperature dependence and equal SRH lifetimes $\tau_{nonrad}$ for electrons and holes. Thus, the SRH recombination rate $R_{SRH}$ is described by

(1) $$R_{SRH}=\frac{np-n_{i}^{2}}{\tau_{nonrad}\left(n+n_{i}\right)+\tau_{nonrad}\left(p+p_{i}\right)}$$

where the defect level is energetically situated at the intrinsic level and $n_{i}$ denoting the intrinsic carrier density. The temperature dependence of the SRH lifetime was modeled via [7]

(2) $$\tau_{nonrad}\left(T\right)=\tau_{0,nonrad}{\left(\frac{T}{300K}\right)}^{-a}$$

The pre-factor $\tau_{0,nonrad}$ denotes the temperature-independent part of the SRH lifetime. The temperature dependence in (2) arises from the temperature dependence of the thermal velocity with $v_{th}\propto T^{0.5}$ and from $\sigma =\sigma \left(T\right)$ in the case of SRH recombination ($\tau_{nonrad}={\left(v_{th}N_{t}\sigma \right)}^{-1}$, where $N_{t}$ is the defect density). Maiberg et al. proposed a temperature dependence of σ∝T, due to harmonic oscillations at finite temperatures [7]. In that case, the exponent a in equation (2) takes a value of 1.5. In a few simulations the exponent was set to a=0, where no temperature dependence was included in the SRH recombination and the transients were simulated at 300 K. The same mobility μ has been set for electrons and holes and is specified for each simulation.

The optical generation was calculated by RayTracing with an absorption coefficient of $8.12\;\mu m^{-1}$ as measured for a CuInSe_2_ absorber [19] and used in previous studies [16]. Thus, in the simulations, the excitation of electron-hole pairs is independent of the GGI grading in the CIGS_front_ region, which simplifies a comparison of the simulated PL decay curves. It is noted that the choice of absorption coefficient mainly affects the initial non-exponential decay characteristics, but not the mono-exponential decay tail time.

No CdS buffer layer was included in the simulations. However, the front surface recombination velocity was varied in order to model the various surface configurations (see above).

The decay time τ of the simulated PL decay curves is calculated according to

(3) $$\frac{1}{\tau }=-\frac{\partial \;ln\left(Y\right)}{\partial t}$$

where Y denotes the time-dependent PL yield. Thus, for a single exponential decay, τ is expected to be time-independent. In the following analysis presented in section 3, the lifetime τ is systematically extracted at a time, where *Y* is reduced by a factor 100 from its maximum value (directly after the excitation pulse) to ensure low injection conditions (see discussion below). This procedure is exemplified in Supplementary Figure 2.

**Figure 2.**
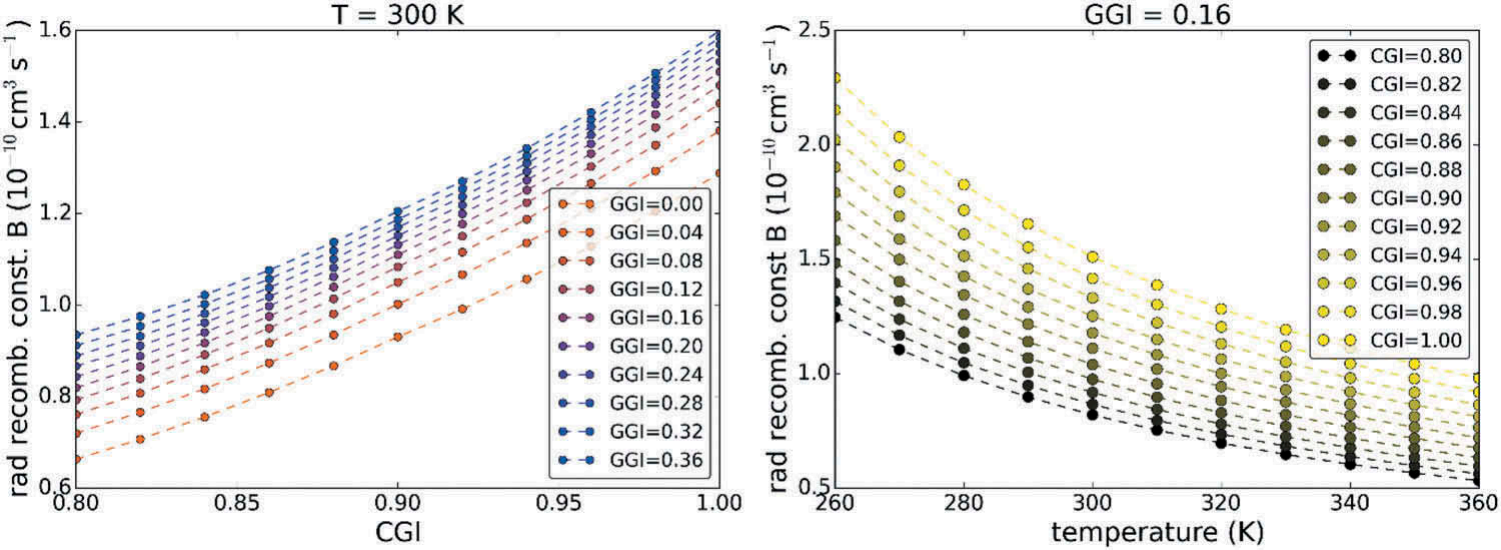
Calculated radiative recombination constants with respect to the CGI and the GGI of a CIGS material (a). In (b) the temperature dependence of the radiative recombination constant is shown for a fixed GGI of 0.16.

The doping density is measured on finished solar cell devices grown in the same deposition run by capacitance voltage (CV) measurements. The modulation frequency was 1 kHz and the apparent doping is extracted at the apparent depth corresponding to the notch of the absorber.

## 3. Results

### 3.1 Calculation of the radiative lifetime

In order to describe the radiative recombination rate $R_{rad}$, the radiative recombination constant B needs to be calculated. This constant is then used for TCAD simulations in section 3.2 as well as the calculation of the external radiative efficiency in section 4.2.

The net radiative recombination rate $R_{rad}$ is given according to

(4) $$R_{rad}=B\left(np-n_{0}p_{0}\right)$$

where n and p denote the electron and hole density, respectively, and $n_{0}$ and $p_{0}$ their equilibrium densities. The net radiative recombination constant B was calculated according to the van Roosbroeck-Shockley relation [20,21]

(5) $$B=\frac{1}{n_{i}^{2}}\frac{8\pi }{h^{3}c^{2}}\overset{\infty }{\underset{0}{\int }}dE\;n_{r}^{2}\alpha \left(E\right)exp\left(-\frac{E}{kT}\right)E^{2}$$

where h the Planck constant, c the speed of light in vacuum, $n_{r}$ the refractive index, α the absorption coefficient, k the Boltzmann constant, T the temperature and E the energy. For the calculation of $n_{i}$ the effective masses are assumed to be $0.1m_{e}$ and $0.9m_{e}$ for electrons and holes, respectively, as in Ref [22], where $m_{e}$ denotes the electron mass in vacuum. The absorption coefficient above the bandgap was determined by transmission and reflection measurements [19] and the real part of the refractive indices are taken from Ref [23]. A bandtail with an Urbach energy of 17 meV was included in the calculation, which describes the absorption below the bandgap. The calculated values for B are shown in Figure 2(a) for variations of GGI and [Cu]/([Ga]+[In]) (CGI). A dependence of B with respect to the CGI originates from higher absorption coefficients for those compositions [19]. Only a minor dependence of B with the GGI value is observed, which can be understood as follows. The factor $n_{i}^{-2}$ depends exponentially on the bandgap (and, therefore, on the GGI). However, the main contribution of the integrand to the integral is also around the bandgap, due to the exponential decrease for higher energies and a strongly decreasing α for lower energies than the bandgap. Hence, the exponential dependence of the bandgap mainly cancels out. In Figure 2(b), the temperature dependence of B is plotted and shows only a weak temperature dependence. As discussed for the bandgap dependence above, the temperature dependence in the exponential terms mainly cancels out with the temperature dependence of $n_{i}^{2}$ and only a weak temperature dependence remains.

By excitation with a laser pulse excess electron-hole pairs are generated with density Δn. For low injection conditions, i.e. $\Delta n\ll p_{0}$, Eqn. (4) reduces to

(6) $$R_{rad}=Bp_{0}\Delta n=\frac{\Delta n}{\tau_{rad}}$$

where the radiative lifetime can be written as

(7) $$\tau_{rad}={\left(Bp_{0}\right)}^{-1}$$

For the TCAD simulations presented in the next section, the radiative recombination constant was set to $B=1.28\times 10^{-10}\;cm^{3}s^{-1}$ (at 300 K), which is obtained for the compositional values in the notch of the CIGS absorber (presented in section 3.3). The doping density was set to$\;p_{0}=3\times 10^{15}cm^{-3}$ as determined by CV measurements (see Supplementary Figure 1). Thus, a radiative lifetime of $\tau_{rad}=2.6\;\mu s$ is obtained.

### 3.2. TCAD simulations

**Figure 3.**
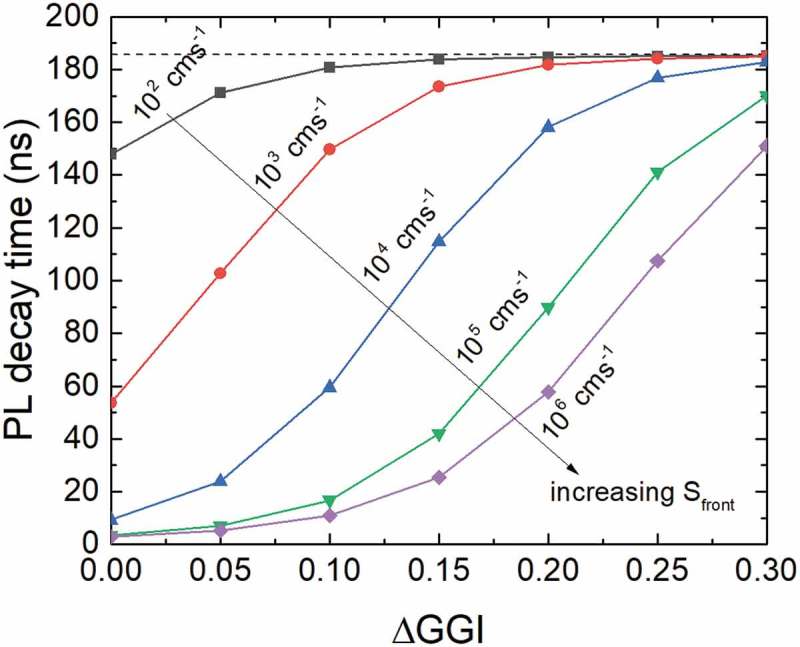
Impact of the increase of the ΔGGI toward the front (see Figure 1) and the front surface recombination velocity $S_{front}$ on the lifetime of the decay curve. The mobility was set to 30 $cm^{2}V^{-1}s^{-1}$ and $\tau_{0,SRH}$ to 200 ns.

Transients with varied front surface recombination velocity $S_{front}$ and ΔGGI were simulated. The decay times were extracted as described in section 2 (see also Supplementary Figure 2) and are plotted in Figure 3. It is observed that for high ΔGGI and low $S_{front}$, the extracted decay times saturate at 185.7 ns (dashed black line), which is expected from the input parameter $\tau_{0.nonrad}=200\;ns$ and thus $\;\tau_{0.nonrad}^{-1}\;+\tau_{rad}^{-1}={\left(185.7\;ns\right)}^{-1}$. Hence, SRH and radiative recombination are the dominant recombination mechanisms for high ΔGGI and low $S_{front}$ values. For lower ΔGGI or higher $S_{front}$, the extracted decay time decreases due to an increased contribution of front surface recombination.

**Figure 4.**
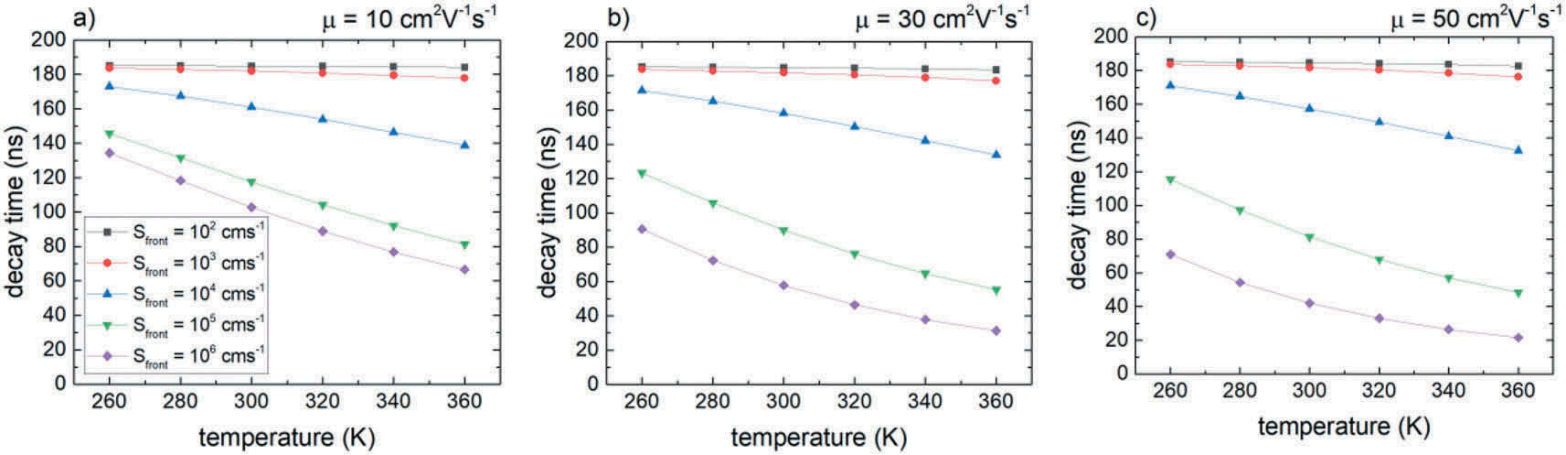
Temperature dependence of extracted lifetimes for various surface recombination velocities and carrier mobilities of $10\;cm^{2}V^{-1}s^{-1}$ (a), $30\;cm^{2}V^{-1}s^{-1}$ (b), and $50\;cm^{2}V^{-1}s^{-1}$ (c). The ΔGGI toward the front contact was set to 0.2 for all simulations and no SRH temperature dependence has been included. No temperature dependence of the SRH recombination has been taken into account. For higher temperatures, an increased emission of electrons over the conduction band barrier toward the front surface results in increased (front) surface recombination.

In particular, the conduction band increase toward the front surface due to ΔGGI imposes a barrier for electrons to recombine at the front surface and hence a temperature dependence of the contribution of front surface recombination is expected. Figure 4 shows the temperature dependence of the extracted decay times for various $S_{front}$ and three different carrier mobilities. For this simulation, no temperature dependence for the SRH recombination has been used, i.e. a=0 (see equation (2)). Thus, the temperature dependence stems from front surface recombination only. The higher the values for $S_{front}$ and μ, the stronger is the temperature dependence of the decay time, which is in accordance with the diffusion theory over a potential barrier [24]. For small$\;S_{front}$, no decrease of the decay time is observed for higher temperatures as surface recombination of electrons emitted over the conduction band barrier does not significantly contribute to the total recombination.

**Figure 5.**
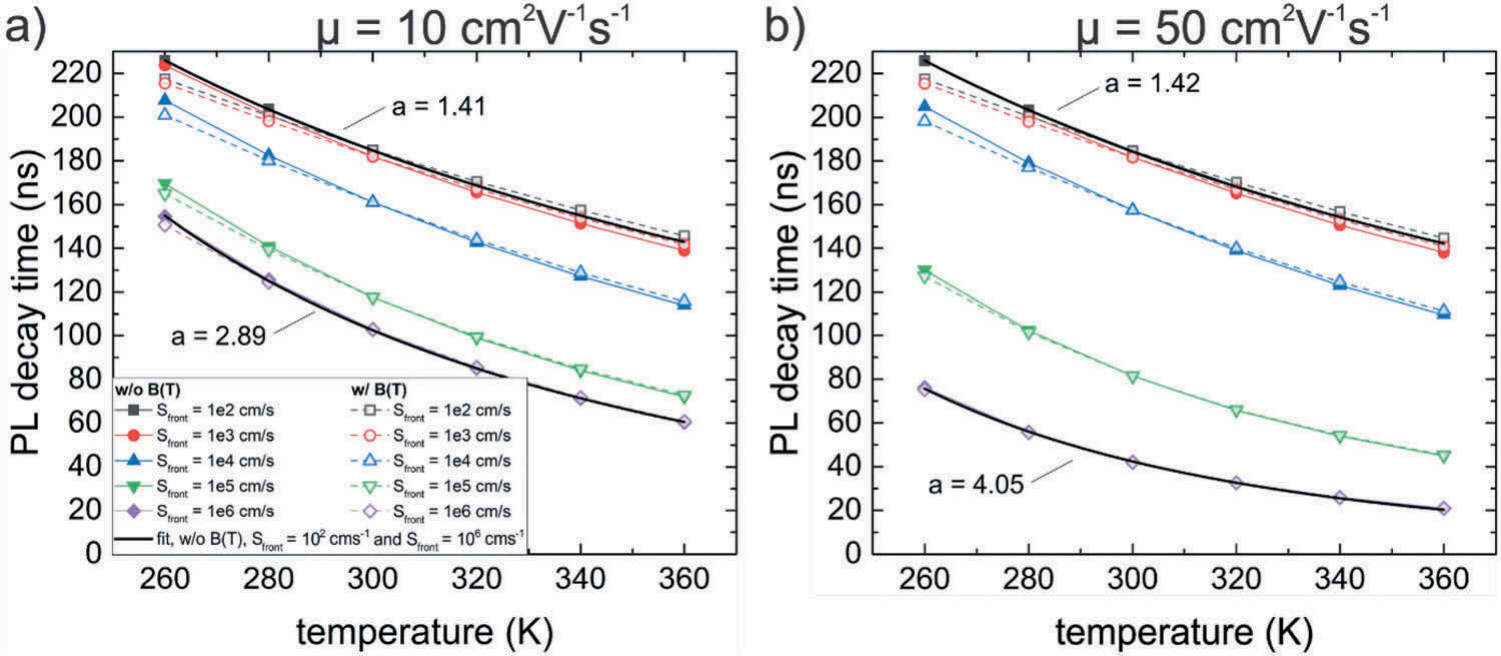
Temperature dependence of the simulated PL decay time without (colored solid lines, full symbols) and with (colored dashed lines, open symbols) a temperature dependence of the radiative recombination constant. The bulk lifetime was set to $\tau_{0,nonrad}=200\;ns$ and ΔGGI=0.2 . Solid black curves are fits to equation (2) and the parameter a describing the temperature dependence is indicated.

Figure 5 shows the extracted PL decay times (from simulated PL transients) with respect to temperature including the SRH temperature dependence by setting a=1.5 (see section 2) in the simulations. The simulations were carried out with (solid lines, full symbols) and without (dashed lines, open symbols) a temperature dependence of the radiative recombination constant (see Eqn. (5) and Figure 2). A small difference for these two cases is observed at lowered temperatures, where the radiative lifetime decreases due to an increasing value for B. At the same time $\tau_{SRH}$ increases and consequently leads to a more significant contribution of the radiative recombination channel. As a result the simulated PL decay times differ significantly when using a temperature-dependent/-independent radiative recombination constant. However, for increased temperatures (as used experimentally), this difference is almost absent. Fitting the simulated PL decay times without a temperature-dependent B (solid lines, full symbols) and for small $S_{front}=10^{2}\;cms^{-1}$ using Eqn. (2) results in an exponent of a ∼1.4. The difference compared to the value used for the simulations (*a* = 1.5) is due to a contribution of radiative recombination. Fitting the temperature dependence in the case of a high value for $S_{front}$ results in a significant higher exponent a of 2.89 and 4.05 for a mobility of 10 and $50\;cm^{2}V^{-1}s^{-1}$, respectively.

SRH recombination cannot explain such a higher temperature dependence and hence another factor influences the recombination dynamics, which is the transport over the front conduction band grading. The higher values of a originate thus from an increased front surface recombination at elevated temperatures. The value of 4.05 for the high mobility case ($50\;cm^{2}V^{-1}s^{-1}$) indicates that diffusion is a significant factor for the transport over the conduction band barrier with a thickness of 0.3 μm (thickness of the CIGS_front_ region, see Figure 1), i.e. as predicted by the diffusion theory [24].

It is noted that the data shown in Figure 5 include radiative recombination. The fits (black curves) on the other hand only take into account SRH recombination (see Eqn. (2)). However, as the radiative lifetime is much higher than the measured lifetime the error is not significant (see discussion above) as the contribution of front surface recombination will have a much stronger impact.

### 3.3. Analysis of experimental TRPL decays

**Figure 6.**
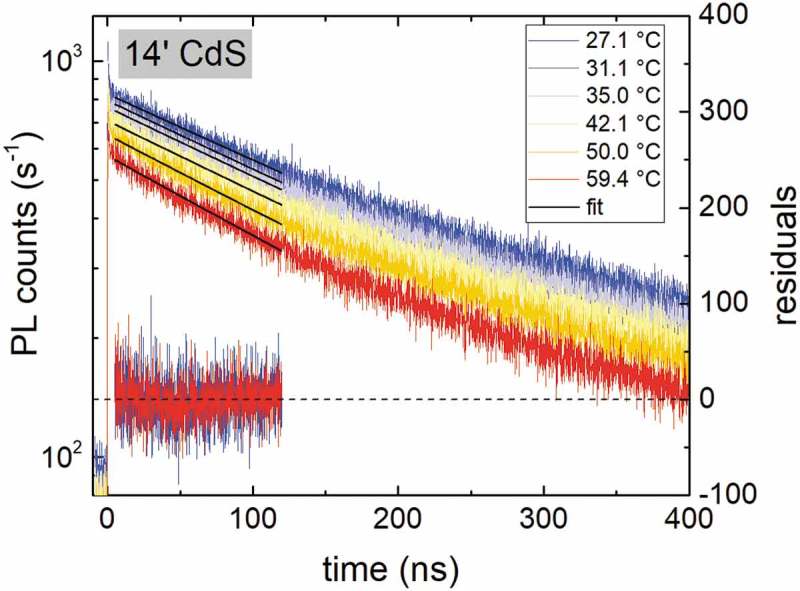
Experimental PL transients with respect to temperature. Single exponential functions were fitted between 5 and 100 ns (solid black lines) to extract the lifetime. The residuals of the fit for the lowest and highest temperature are plotted on the right ordinate indicating a single exponential behavior in this time range.

**Figure 7.**
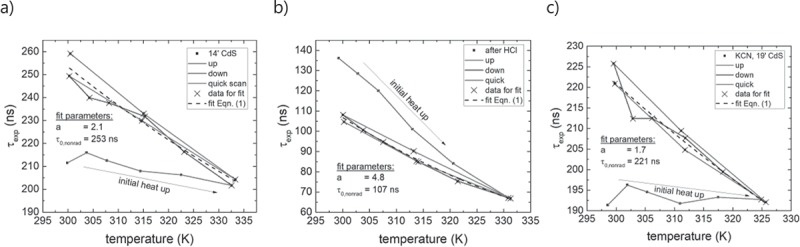
Extracted lifetimes from the transients with respect to temperature of the 14ʹ CdS sample (a), the HCl etch sample (b) and the KCN etched with a 19ʹ CdS layer (c).

Figure 6 shows experimental PL transients for the 14ʹ CdS sample measured from high to low temperatures (first cool down cycle). With decreasing temperature, the PL decay time increases as expected from the SRH recombination as well as front surface recombination in combination with a double-graded CIGS absorber. The PL lifetimes were extracted using a single exponential fit within 5 and 100 ns (black dashed lines). The residuals are plotted on the right ordinate indicating a single exponential behavior in this time range. Fitted PL decay times are plotted with respect to temperature for the *14ʹ CdS* sample in Figure 7a.

During the initial heat up of the sample (red curve), the decay times show only a minor temperature dependence. This minor temperature dependence is due to an improving quality (less recombination) of the sample under test (absorber covered with *14ʹ CdS*). In fact, upon switching on the pulsed laser, the count rate increases with prolonged exposure of the laser as a result of an increasing lifetime (not shown). Hence, during the initial heat up, the sample quality is not stable. The improved quality is evident during the cool down (blue line), which shows significantly longer decay times when compared to the heat up (red line). A second, faster temperature sweeping cycle verifies the stable sample state (green line) yielding similar lifetimes as during the first cool down. To extract the parameter *a* for the temperature dependence, only data points acquired from a stable sample quality (black crosses) have been used for the fitting with Eqn. (2). The fitted values are $\tau_{0,nonrad}=253\;ns$ and a=2.1. It is noted that the radiative recombination is not included in this analysis due to an estimated radiative lifetime of $\tau_{rad}=2.6\;\mu s$, which is much larger than PL decay times. A further discussion is given in section 4.2.

The same measurement and analysis procedure has been carried out for the two other configurations ‘*after HCl*‘ and ‘*KCN, 19ʹ CdS*’ and are presented in Figures 7(b) and (c). Upon the initial heat up the *after HCl* sample shows a more pronounced temperature dependence compared to the cool down. The second temperature cycle (green line) verifies a stable state and indicates a degradation of the sample quality. This can be assigned to a degradation of the free CIGS surface due to exposure to ambient air [10,17]. Fitting the data points measured for a stable sample quality (black crosses) yields values of a=4.8 and $\tau_{0,nonrad}=107\;ns$. The strong temperature dependence indicated by the exponent a=4.8 can be ascribed to a highly recombinative front surface as shown in section 3.2. This is consistent with the lowered value for $\tau_{0,nonrad}$, which in this case is influenced by front surface recombination and does not represent the bulk absorber lifetime. Principally, modified bulk properties with an electron trap could also explain such a strong temperature dependence [7], which might be introduced during the exposure to ambient air at elevated temperatures. However, the sample *KCN, 19ʹ CdS* yields similar parameters of $\tau_{0,SRH}=221\;ns$ and a=1.7 (Figure 7(c)) as the initial *14ʹ CdS* sample and thus hints mainly to a modification of the front interface properties instead of altered bulk properties.

## 4. Discussion

### 4.1. Trapping of minority charge carriers

In section 3.2 and 3.3, it was demonstrated that a strong temperature dependence of the PL decay time can be the result of a highly recombinative front surface in combination with a front conduction band grading (due to a Ga front grading). From the measurements with a CdS-covered front surface, only a low temperature dependence is observed, which is described with the exponent in Eqn. (2) of a=1.7−2.1 (Figure 7). For the simulations of PL transients presented in section 3.2, the value of a is assumed to be 1.5, where the contribution of the capture cross-section contributes with σ∝T. However, also a stronger temperature dependence of σ with an exponent >1 can be applied [7]. Thus, the experimental values of a slightly bigger than 1.5 can be explained by the temperature dependence of the SRH recombination and no electron traps need to be implemented in order to correctly describe the transients. In addition, the front surface recombination velocity for a CdS-covered surface must be small, since a higher temperature dependence would be observed otherwise, consistent with previous TRPL studies (without a front Ga front grading) [16].

In the case of a high $S_{front}$ (*‘after HCl’*), the experimental temperature dependence is fitted with a=4.8, which is a rather large value when compared to the simulations (Figure 5) and hints to a rather high mobility with $\mu >50\;cm^{2}V^{-1}s^{-1}$ (in the simulations an exponent of a=4.05 has been determined with the parameters $\mu =50\;\;cm^{2}V^{-1}s^{-1}$ and ΔGGI=0.2). Values of $\mu >55\;cm^{2}V^{-1}s^{-1}$ were previously determined by spectrally resolved TRPL measurements [25] and are thus consistent with a rather large value for the exponent a in the case of a high $S_{front}$. It is noted that the ΔGGI for the sample under investigation is only around 0.1 as can be observed from the GGI grading shown in Supplementary Figure 1. Simulations with ΔGGI=0.1 and $\mu >50\;cm^{2}V^{-1}s^{-1}$ resulted in exponents of a not exceeding the value of 3. However, the band profile for the simulations is quite simplified and changes of the bandgap (and thus potentially the conduction band edge) might also result from a Cu depletion at the surface [26,27] or the formation of alkaline phases [18,28,29]. These effects were not considered in the TCAD model and might induce an additional barrier for the electrons to recombine at the front surface and consequently result in a faster decrease of the PL decay time with increasing temperature for the *‘after HCl’* configuration, i.e. a recombinative front surface.

Another feature observed in experiment is a fast initial decay within the first few ns after the laser excitation pulse. A zoom of the transients shown in Figure 6 to the first 30 ns is presented in Supplementary Figure 3. Such an initial transient with a very small decay time can be attributed to trapping as a consequence of a reduction of minority carriers on a short time scale. However, as mentioned in section 2, the excitation density was in the same order as the doping density and, therefore, the transient within the first few ns is strongly influenced by high excitation effects such as bi-molecular recombination and diffusion into the notch and can also be reproduced by the simulations without trap states (Supplementary Figure 3). Decreasing the excitation density experimentally and performing the measurements strictly in low excitation conditions should yield more information about the origin of the fast initial decay. However, the sample under test had a doping density of ~$3e15\;cm^{-3}$ and the TRPL setup used in this study could not be operated within the corresponding low excitation conditions.

In conclusion, for the CIGS absorber investigated in this study, all effects, which previously were assigned to trapping [7] such as a fast initial decay and a strong temperature-dependent PL decay time can also be explained when taking into the account the band structure of a double-graded CIGS absorber layer. However, trapping effects of the measured PL transients cannot be excluded at this point. In reference [7], the temperature dependence of the PL decay time was more pronounced (decrease of τ by a factor of 4 when increasing the temperature from 300 K to 330 K) compared to the samples in this study (decrease by a factor of 1.5). Hence, the magnitude of trapping might depend on the absorber preparation. Werner et al. found a donor-like defect state 100 meV below the conduction band, whose density is dependent on the process conditions (in that case the Cu-supply during growth) [30] and could be responsible for the trapping effects in [7].

Assuming that the complete temperature dependence of the PL decay time results from SRH recombination, the PL decay time corresponds to the minority carrier lifetime in the notch region of the CIGS absorber if the front surface recombination velocity is not too high (compare Figure 3).

### 4.2 Photoluminescence efficiency

In section 4.1, it has been shown that the temperature-dependent TRPL measurements can be explained by the band structure of a double-graded CIGS absorber and without trapping. In that case, the PL decay time corresponds to the bulk recombination time of minority charge carriers in the notch region of the CIGS absorber. Based on these assumptions, the internal radiative efficiency $\eta_{int}$ can be calculated according to [21]

(8) $$\eta_{int}=\frac{1}{1+\tau_{rad}/\tau_{nonrad}}=\frac{\tau_{eff}}{\tau_{rad}}$$

with

(9) $$\frac{1}{\tau_{eff}}=\frac{1}{\tau_{rad}}+\frac{1}{\tau_{nonrad}}$$

Using a radiative lifetime of 2.6 μs and an effective lifetime of 253 ns (Figure 7(a)) an internal radiative efficiency of approximately 10% is obtained (at 300 K).

The external PL efficiency $\eta_{ext}$ can be defined as [31]

(10) $$\eta_{ext}=G^{\prime }\frac{\tau_{eff}}{\tau_{rad}}=G^{\prime }\eta_{int}$$

where $G^{\prime }$ is the fraction of photons, which escape from the sample front surface. Using $n_{CIGS}=3$ and $n_{air}=1$ and following the approach of [32], $G^{\prime }$ evaluates to

(11) $$G^{\prime }=\frac{1}{4n_{CIGS}^{2}}\left(1-R\right)=\frac{1}{4n_{CIGS}^{2}}\left[1-{\left(\frac{n_{CIGS}-n_{air}}{n_{CIGS}+n_{air}}\right)}^{2}\right]\approx 0.021$$

Thus, only around 2.1 % of the photons generated by radiative recombination escape the CIGS layer in the detection direction, resulting in an external PL efficiency $\eta_{ext}$ around 0.21%. The first term in Eqn. (11) accounts for total reflection at the front interface for photons with an angle of incidence larger than the critical angle. The second term in the square brackets is the transmission probability for photons with an angle smaller than the critical angle. The reflection at the back contact has been neglected due to the small reflectivity of the CIGS/Mo interface [33,34].

Measuring the absolute PL counts from the CIGS absorber yields an external PL efficiency of$\;\eta_{ext,PL}=0.17\%$. This direct measurement of the PL efficiency is in good agreement with the value obtained from TRPL decay times and Eqn. (11) resulting in a value of 0.21%. As a consequence, the measured decay time can indeed be assigned to the bulk minority carrier lifetime, which is not influenced by minority carrier trapping.

### 4.3 Comparison of the QFLS

The TRPL decay time can be used to calculate the QFLS $\Delta \mu_{TRPL}$ of the absorber and will be compared to the QFLS $\Delta \mu_{PL}$ measured by absolute PL measurements at the end of this section. In order to be comparable between these two techniques, the generation rate of electron-hole pairs for the calculation of $\mu_{TRPL}$ is estimated based on the photon flux from the absolute PL measurement. For that purpose, the photon flux of $2.76\times 10^{17}cm^{-2}s^{-1}$ is corrected for reflection at 660 nm, which was measured to be 13%. The resulting generation rate is $G_{TRPL}=2.4\times 10^{17}\;cm^{-2}s^{-1}$. Thus, at any instant, the number of excess charges per unit area Δq in the absorber is:

(12) $$\Delta q=G_{TRPL}\times \;\tau_{TRPL}$$

In order to calculate the QFLS to realize the charge Δq of electrons in the conduction band and holes in the valence band, the compositional grading needs to be taken into account and is done as follows. The GGI grading determined from SIMS measurements (see Supplementary Figure 1) is discretized into layers with thicknesses $d_{i}=$ 25 nm. For each layer i, the bandgap $E_{g,i}$ is here determined from the relation $E_{g}=1.004\left(1-GGI\right)+\;1.663\;GGI-0.033\;GGI\left(1-GGI\right)$ [19]. The GGI is assumed to only act on the conduction band, such that for each layer *i* we set the energies for the valence band maximum $E_{v,i}=0$ and for the conduction band minimum $E_{c,i}=E_{g,i}$. The effective density of states for electron and holes are computed from the effective masses $0.1m_{e}$ and $0.9m_{e}$ as mentioned above.

Then, the values for the quasi-Fermi levels for electrons $E_{fn}$ (Equation (13)) and holes $E_{fp}$ (Equation (14)) are determined, such that the charge carriers in the bands match the number of excess charges Δq plus their intrinsic charges ($n_{0,i}$ for electrons and $p_{0,i}$ for holes).

(13) $$\Delta q+\underset{layers\;i}{\sum }d_{i}\;n_{0,i}=\underset{layers\;i}{\sum }d_{i}\;N_{c}exp\left(-\frac{E_{c,i}-E_{fn}}{k_{B}T}\right)$$

(14) $$\Delta q+\underset{layers\;i}{\sum }d_{i}\;p_{0}=\underset{layers\;i}{\sum }d_{i}\;N_{v}exp\left(-\frac{E_{fp}-E_{v}}{k_{B}T}\right)$$

The doping density $p_{0}$ in the CIGS absorber is assumed constant in each of the layers and is estimated from C-V measurements, while the density of minority charge carriers $n_{0,i}$ is computed in each of the layers from the local bandgap:

(15) $$n_{0,i}\;p_{0}=n_{i}^{2}=N_{C}N_{V}exp\left(-\frac{E_{g,i}}{k_{B}T}\right)$$

Finally, the QFLS $\Delta \mu_{TRPL}$ is obtained from $\Delta \mu_{TRPL}=E_{fn}-E_{fp}$ .

The calculation was performed using as input parameters a doping density$\;p_{0}=3\times 10^{15}cm^{-3}$, $\tau_{TRPL}=250\;ns$ and the GGI profile shown in Supplementary Figure 1. This profile was verified by optical Transfer-Matrix-Method simulations to reproduce the experimental EQE edge [19]. A calculated value of $\Delta \mu_{TRPL}=735\;meV$ was obtained, which matches the quasi-Fermi level $\Delta \mu_{PL}=731\;meV$ extracted from an absolute PL measurement (see Supplementary Figure 4).

This good agreement suggests that the $\tau_{TRPL}$ value acquired on CdS-covered samples is representative for both bulk and interface recombination in the final device and is not dominated by trapping effects. If trapping had a significant influence on the TRPL lifetime, the value for $\tau_{TRPL}$ would be overestimated resulting in an overestimation of Δq and consequently also of $\Delta \mu_{TRPL}$. In contrast, the measured value of the QFLS from a PL measurement $\Delta \mu_{PL}$ would not be influenced by trapping as under continuous wave excitation the occupancy of the trap states is in equilibrium with the injected excess charge carriers.

It is worth noting any error of $p_{0}$ and $\tau_{TRPL}$ on $\Delta \mu_{TRPL}$. The lifetime $\tau_{TRPL}$ directly influences the number of excess charge carriers Δq (see Eqn. (12)), which affects the electron quasi-Fermi level $E_{fn}$ logarithmically via Eqn. (13). Thus, an error of $\tau_{TRPL}$ by a factor of 2 will change $E_{fn}$ by $\ln\left(2\right)\cdot k_{B}T\approx 18\;meV$ (at room temperature). Similarly, a change of the doping $p_{0}$ by a factor of 2 will change the hole quasi-Fermi level $E_{fp}$ by ≈18 meV via Eqn. (14) and low excitation conditions (which is generally met under 1 sun illumination). Consequently, the difference of $\Delta \mu_{PL}$ and $\Delta \mu_{TRPL}$ can be explained by small deviations of the input parameters $\tau_{TRPL}$ and $p_{0}$. In particular, major deviations of $\tau_{TRPL}$ are not expected as for instance reported for CIGS absorbers, which are influenced by trapping [6,35], where the measured decay time is in the order of 100 ns, while the simulation of the decay curve yielded only 1–20 ns.

It is noted that the experimental $V_{OC}$ is only 720 mV and thus slightly below the QFLS. This discrepancy has been observed previously [36] and might suggest an increased recombination upon deposition of the window layers of the solar cell device.

## 5. Conclusions

In this manuscript, the impact of the double-graded CIGS absorber on the temperature dependence of the PL decay time has been demonstrated. In particular, TCAD simulations were carried out, which show that the conduction band increase toward the front contact imposes a barrier for the excess electrons and, therefore, reduces front surface recombination. Consequently, the temperature dependence of the PL decay time increases due to an increased transport of electrons over the front-graded part of the absorber. The higher the front surface recombination velocity, the more pronounced is the temperature dependence of the PL decay time, which was verified by experiments. A CdS-covered CIGS absorber showed experimentally a small temperature dependence of the PL decay time, which could be explained by the temperature dependence of the SRH recombination and without trapping of minority carriers. In contrast, a free CIGS front surface resulted in a stronger temperature dependence due to the increased transport of electrons over the conduction band barrier from the notch to the front surface. This stronger temperature dependence is not due to trapping. Consequently, for investigations of the temperature dependence of the SRH recombination, the double-graded CIGS band structure has to be taken into account or the front surface has to be well passivated. The measured PL decay time for the CdS passivated CIGS absorber in this study yields 250 ns at room temperature and can thus be assigned to the minority carrier lifetime in the notch region. The external radiative efficiency for that lifetime corresponds to approximately 0.2% as calculated from the lifetime measurement and verified by absolute PL measurements. The calculated QFLS based on this minority carrier lifetime is 735 meV and matches well the measured QFLS from an absolute PL measurement. Therefore, it is concluded that the measured lifetime by TRPL is not influenced by trapping of minority carriers and represents the bulk minority carrier lifetime.
