# Supplementary material for: Time-resolved photoluminescence on double graded Cu(In,Ga)Se2 – Impact of front surface recombination and its temperature dependence
Source: Sci Technol Adv Mater. 2019 Apr 9;20(1):313–23. doi: 10.1080/14686996.2019.1586583 (PMC6484473; doi:10.1080/14686996.2019.1586583)
Supplement: Supplemental Material [file TSTA_A_1586583_SM7489.pdf]

Supplementary Material

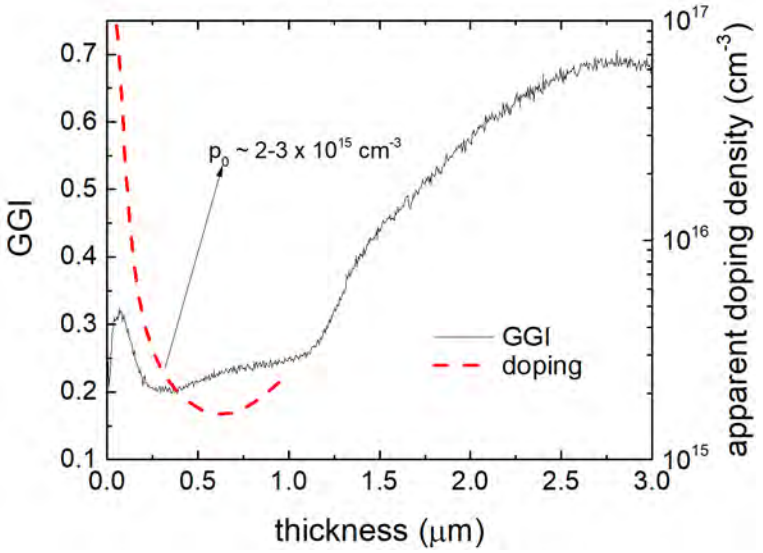

Supplementary Figure 1 – The GGI determined from the SIMS profiling (black curve, left ordinate). The apparent doping density (measured by C(V)) was estimated between $2-3 \times 10^{15} cm^{-3}$ in the notch region (red dashed line, right ordinate).

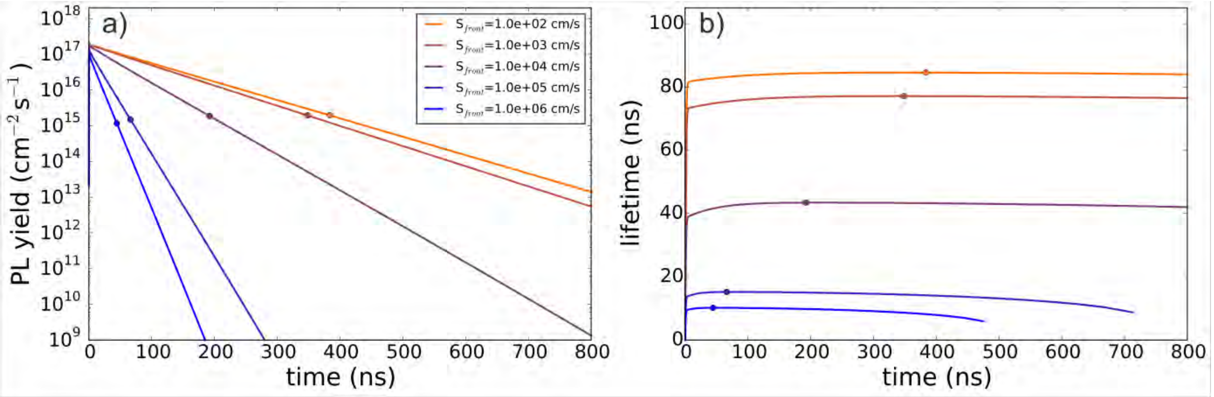

Supplementary Figure 2 – a) Simulated PL transients for a mobility of 30 $cm^2V^{-1}s^{-1}$, a bulk lifetime of $100\ ns$ and a variation of the front surface recombination velocity. The ΔGGI towards the front was set to 0.1. Solid circles indicate, where the PL yield is reduced by a factor of 100 with respect to the maximum PL yield (directly after the excitation pulse). b) The lifetime τ calculated from the derivative of the PL yield Y according to $\frac{1}{\tau} = -\partial ln(Y)/\partial t$.

a) b)

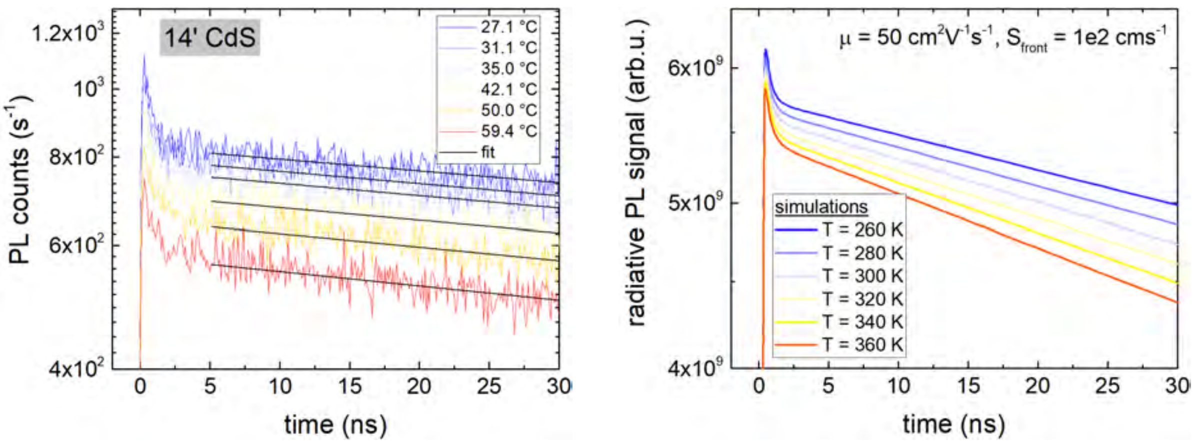

Supplementary Figure 3 – Initial behavior of the PL transient. Directly after the laser pulse a fast decay is observed within the first few ns.

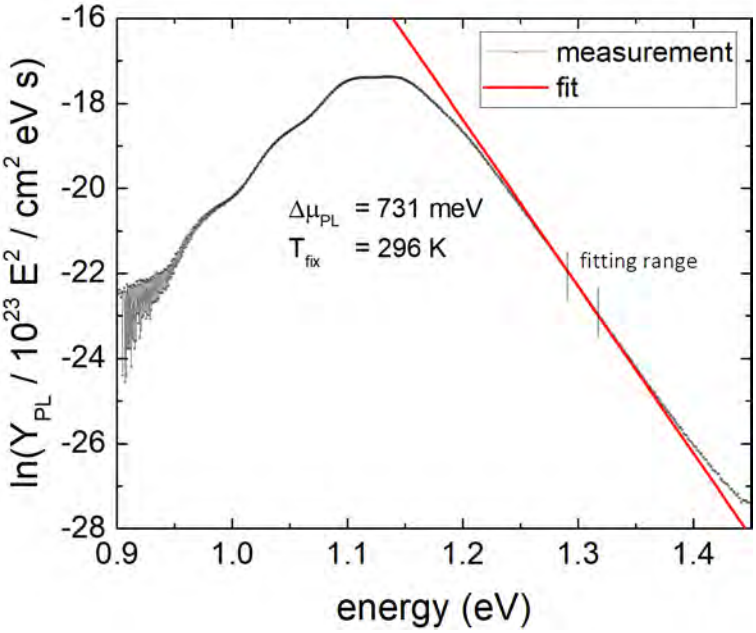

Supplementary Figure 4 – Evaluation of the quasi Fermi level splitting $\Delta\mu_{PL}$ from an absolute PL measurement of the sample in the *14' CdS* configuration. The fitting range was set to a narrow range around 1.3 eV. For lower energies the assumption of $a(E) = 1$ might not be satisfied anymore, while for higher energies residual background is present and hence leads to a change in slope. The temperature has been fixed for the fitting to $T_{fix} = 296\ K$, which corresponds to the temperature of the lab during data acquisition.
